# Brain Metastases in Patients with Gynecologic Cancers: A Single Institution Experience and Review of the Literature

**DOI:** 10.4236/ojog.2016.69070

**Published:** 2016-08-15

**Authors:** Madiha A. Gilani, Noelle L. Williams, Carolyn Giordano, Norman Rosenblum, Wenyin Shi, Pramila Anne, Russell J. Schilder

**Affiliations:** 1 Thomas Jefferson University Hospital, Philadelphia, PA, USA; 2 Department of Radiation Oncology, Sidney Kimmel Cancer Center, Thomas Jefferson University Hospital, Philadelphia, PA, USA; 3 Office of Institutional Research, Thomas Jefferson University, Philadelphia, PA, USA; 4 Sidney Kimmel Cancer Center, Thomas Jefferson University Hospital, Philadelphia, PA, USA

**Keywords:** Gynecologic Malignancies, Brain Metastasis, Prognostic Factors, Multimodality Therapy

## Abstract

**Objective:**

Brain Metastasis (BM) from primary gynecologic cancers is a rare entity. The advances and successes in the treatment of primary gynecologic malignancies, have led to prolonged survival and, a higher incidence of BM. This study aims to report the experience at our institution in managing these patients, and provide possible data points that may be essential to note as prognostic factors, and see if our findings are consistent with the literature in this subject. We also aim to provide a brief literature review of patients with gynecologic cancers and BM.

**Methods:**

This is a small single institution retrospective study of 23 patients with a gynecologic malignancy and BM, identified between the years 2007–2015. Data were collected on variables including patient demographics, disease and treatment.

**Results:**

The median overall survival from the primary diagnosis was 28 months. Median time from diagnosis of BM to death was 9 months.

**Conclusion:**

The outcomes in our study are similar to what is stated in the current literature with regard to BM from gynecologic malignancies. Our literature search also revealed that the molecular analysis and treatment of the primary tumor remain important to prevent BMs. The tendency of tumors to metastasize varies for one tumor type to another for the same type of tumor. The tendency to develop BM may not only depend on risk factors such as stage, grade, and histology, but also on the genetic profile of the primary tumor. The study suggests that multimodal treatment of BM has better outcomes in managing BM from gynecologic cancers.

## 1. Introduction

Brain Metastasis (BM) is a poor prognostic indicator, resulting in reduced survival. It is the most common neurologic complication of cancer. Cancers that commonly metastasize to the brain include lung cancer, breast cancer and melanoma to name a few [1]. The most common histology of metastases found in the brain is adenocarcinoma. Gynecologic malignancies, unlike tumors of the lung, breast and melanoma, less commonly metastasize to the brain. They do so by hematogenous spread. These cancers are usually metastatic to the liver, lungs, lymph nodes and bones. Ovarian cancer has the highest incidence of BM among gynecologic cancers (0.3% - 2.2%), followed by endometrial (0.4% - 1.2%) then cervical cancer (0.3% - 0.9%) [2] [3]. Over 30 years ago, BM from gynecologic cancers was an even more rare event than it is now, since platinum based therapy became the initial standard of care for majority of gynecologic malignancies. Patients in the pre platinum era had shorter survivals and not enough time to develop BM. The hypothesis why this maybe is as follows: 1) Platinum based therapy has shown excellent overall survival results. Still, the incidence of relapse is not negligible, but the duration between relapses is long [4]. Microscopic cancer cells during remission are not detected on surveillance imaging, or by the current available blood tests. This gives the cancer an adequate amount of time to re-establish itself in sites that are difficult to access and hence a challenge to treat, such as the brain. In the presence of disease, the Blood Brain Barrier (BBB), loses its integrity to metastatic disease, which manifests in the brain. Here it is protected from being attacked by the immune system and chemotherapeutic drugs [5]. 2) Radiologic imaging has come a long way, and is more sensitive in detecting small intracranial lesions. Hence BM is easier to find than they were in the past [1]. 3) The ease of availability of high quality imaging, such as MRI to clinicians, contributes to early detection of BM today.

In our literature search, we came across no prospective studies on brain metastasis in patients with gynecologic malignancies. All are retrospective studies and case reports that have tried to describe the experience of managing BM and possible risk factors that may lead to BM from gynecologic cancers. These studies have taken place over different periods of time as oncology has evolved, and newer and better treatment modalities were being introduced in medical oncology, radiation oncology and radiology. These studies demonstrate that over time the incidence of BM from one study to the other not only increased, but the survival from BM improved as well, in this setting.

Given the rare occurrence of brain metastasis from a gynecologic primary source, there are no specific guidelines in place to anticipate their presence or to treat these lesions. In majority of the cases with BM, scans of the chest, abdomen and pelvis, done for surveillance purposes post initial chemotherapy, are negative for new or metastatic disease. In spite of a detailed history and clinical examination during each visit, BM frequently escapes recognition. Commonly the finding is acute, when the patient presents to an emergency room with a subtle or obvious neurologic deficit, leading to imaging of the brain that demonstrates the lesion as a consequence of the underlying gynecologic malignancy. As survival improves, CNS disease from gynecologic cancers is likely to become more prevalent.

## 2. Materials and Methods

This is a single institution, retrospective study, with a primary goal to present our experience with managing patients who have an underlying gynecologic primary cancer, and BM. The secondary goal is to try and identify risk factors that may predispose patients to developing BM, and also to identify prognostic factors that may impact survival in these patients.

### 2.1. Patient Characteristics

From January 2007 to December 2015, we identified 23 patients with a primary gynecologic cancer and BM at Thomas Jefferson University Hospital in Philadelphia. Patients that were included in the study had an underlying gynecologic malignancy, diagnosed by imaging and or histopathology. Most had received initial therapy at our institution, but some were referred to us for treatment of BM, following initial therapy for the primary tumor elsewhere. We did not have the initial pathologic or treatment data of the primary tumor for this group of patients. Diagnosis of primary tumors made at our institution were based on a combination of radiologic imaging, tissue specimens obtained via biopsy or surgery, use of the appropriate immunohistochemical stains, and for ovarian cancer also included a serum level of CA125. For BM, histopathologic diagnosis of was not necessary for the study. BM was diagnosed clinically and based on MRI of the brain. Exclusion criteria included history of a concomitant second malignancy or history of a primary brain tumor that was treated I the past.

A retrospective chart review was conducted of the medical, surgical, radiologic and histopathalogic record of each patient. IRB approval was obtained to perform a retrospective chart review to collect the following data: age at diagnosis of the primary tumor, date of diagnosis, histology, grade based on the International Federation of Obstetricians and Gynecologists (FIGO) grading system, presence or absence of lymphovascular invasion, sites of metastasis other than the brain, FIGO stage of the disease, CA125 at the time of diagnosis in the case of ovarian cancer and at the time of diagnosis of BM, type of treatment received for the primary tumor, date of diagnosis of brain metastasis, time between diagnosis of the primary tumor and development of brain metastasis, number and size of metastasis, symptoms if present, KPS at the time of initial diagnosis of the BM, treatment of BM, molecular analysis of primary or metastatic lesion, last follow up appointment with medical oncology or radiation oncology and if the patient was dead or alive by the time this retrospective study was concluded. The Recursive Partitioning Analysis (RPA) class was calculated for each patient at the time of diagnosis of BM.

### 2.2. Treatment of the Primary Tumor

Majority of the patients with primary ovarian or endometrial cancer underwent optimal debulking surgery, and no gross residual disease remained post operatively. One patient received neoadjuvant treatment prior to surgery due to the extent of the disease at the time of initial diagnosis. The rest of the patients with ovarian and endometrial cancer received adjuvant chemotherapy with carboplatin and paclitaxel. Majority received the dose dense regimen. There were 2 patients with Stage II cervical cancer, who received cisplatin and brachytherapy. Information on treatment of the primary cancer was not available on 4 patients with ovarian cancer, 1 with cervical, 1 with uterine sarcoma and 1 with vulvar cancer. Patients with rare and aggressive subtypes such as small cell carcinomas and sarcomas are discussed separately.

### 2.3. Treatment of Brain Metastasis

10 patients (43.4%) had neurosurgical resection at initial diagnosis of BM. 15 of the 23 patients received whole brain radiotherapy (WBRT). 3 patients who received WBRT had a prior resection. Of the 7 patients receiving stereotactic radiosurgery (SRS), 3 patients had 1 target (42.9%), 3 patients had 2 targets (42.9%), and 1 patient had 3 targets (14.2%). Two of the patients having received WBRT (13.3%) had a planned SRS tumor boost following completion of WBRT. One patient received fractionated stereotactic radiotherapy (FSRT) given the size of the surgical cavity. WBRT technique was opposed lateral fields with a dose ranging from 30 Gy in 10 fractions to 37.5 Gy in 15 fractions. Eight patients received 30 Gy (53.3%), 6 received 37.5 Gy (40.0%), and 1 received 35.5 Gy (6.7%) with fractionation that transitioned over the treatment course to shorten duration of therapy.

## 3. Statistical Analysis

Statistical analysis was performed using SPSS v 22 (IBM Corp. Released 2013. IBM SPSS Statistics for Windows, Version 22.0. Armonk, NY: IBM Corp.).

This is a retrospective chart review and a descriptive account of our experience at Thomas Jefferson University Hospital. The data are presented as the mean and median for survival by cancer type. Kaplan-Meier survival curves were generated to evaluate survival functions by type of cancer.

Given that the overall number of patients is very small, this is primarily a descriptive study and no inferential statistics were computed.

## 4. Results

As described in Table 1, 23 patients were collected from our database with a primary gynecologic malignancy and BM (One patient had both Stage I ovarian and Stage I endometrial cancer). The mean age at diagnosis of the primary cancer was 60 years (46 – 79 yrs). 15 (65%) of the patients were Caucasian, 3% (13%) were non-Caucasian. The racial origin of 5 (22%) was unknown. The mean time from the diagnosis of the primary tumor to the development of brain metastasis was 27 months (0 – 83 months), and specifically 31, 22, 25, and 26 months for ovarian, endometrial, cervical and vulvar cancer respectively. Median time from initial diagnosis to BMD was 18 months (range 3 – 82). The mean age at time of brain metastasis diagnosis was 62 years (range 49 – 81). 13 (57%) patients had primary ovarian/fallopian tube cancer, 6 (26%) had endometrial cancer, 13% (3) had cancer of the cervix, and 1 (4%) had vulvar cancer. Of the 13 (57%) patients with ovarian cancer, 9 (69%) patients had adenocarcinoma, and 5 of these were serous type. We were unable to obtain the data on 4 patients with regards to the specific histology of ovarian adenocarcinoma. Of the patients with ovarian carcinoma, 1 patient had ovarian carcinosarcoma and 3 patient’s specific histology was unknown. Of the 6 patients with endometrial cancer, 3 (50%) had adenocarcinoma, and 3 (50%) had uterine sarcoma. Of the 4 patients with cervical cancer, 50% (2) had small cell cancer and 50% (2) had squamous cell carcinoma. The 1 (4%) patient with vulvar cancer had small cell cancer and this was also the only patient with Stage IV disease. 11 (48%) patients had FIGO Stage III disease. Of these, 9 had ovarian cancer and 2 had endometrial cancer. 3 (13%) patients had cervical cancer and these were the only patients with Stage II disease. 2 (33%) endometrial cancers and 1 (8%) ovarian cancer had Stage I disease. Amongst these three tumors, 1 patient had synchronous Stage I ovarian and endometrial cancers. Initial staging of the primary tumor was unknown in 6 (25%) patients, including 3 (23%) with ovarian cancer, 2 (33%) with endometrial and 1 (25%) with cervical cancer. FIGO grading was available for 8 (34%) patients, including 3 patients with grade 2, and 5 patients with grade 3. There were no patients with grade 1 disease. Of the patients with grade 2, one patient had endometrial, cancer, 1 had ovarian cancer and 1 patient had both endometrial and ovarian cancer. Of the 5 patients with grade 3 tumors, all had ovarian carcinoma. In the patient who had received neoadjuvant chemotherapy for ovarian carcinoma, no assessment could be made on the FIGO grading of this patients primary tumor. 6 of the 23 patients demonstrated lymphovascular invasion (LVI) and this was unknown in the rest. The CA 125 was known in 6 out of 13 patients with ovarian cancer. The mean CA 125 at the time of initial diagnosis of ovarian cancer was 797.2 U/ml, and the mean CA 125 at the time of diagnosis of brain metastasis in the same patients was 43 U/mL.

Approximately 8 (34%) of the patients had relapsed with extra-cranial disease around the time they were diagnosed with BM. 2 patients had lung metastases earlier during the disease course prior to being diagnosed with brain metastasis. Of these, 1 was the patient with Stage IV small cell vulvar cancer, and the other had primary endometrial cancer of endometroid type. The latter patient had oligo metastases to the lung, which were removed soon after the diagnosis of the primary tumor. Overall, sites of extracranial disease at the time of diagnosis of BM included 1 patient with mediastinal, 1 with right axillary, 1 with liver, 3 with lung and 1 patient with both liver and lung metastasis.

All patients in this series presented with symptoms prior to diagnosis of brain metastasis. Most frequent symptoms reported were headache, ataxia, confusion and weakness. 15 (65.2%) patients had less than 3 BM, 7 (30.4%) had 3 or more BM, and 1 (4.2%) had leptomeningeal disease at the time of diagnosis of disease in the brain.

10 (43.5%) patients were of RPA class 1, 10 (43.5%) of RPA class 2, and 3 (13.0%) of RPA class 3. From the time of diagnosis of brain metastasis the patients were followed for a mean time period of 13.5 months. The duration of follow up for 1 patient is unknown as she is still alive and went to another facility.

## 5. Molecular Analysis

Tumors in 5 (21%) patients were sent for molecular analysis. We took account of this data to look for mutations that might be common to these tumors, and possibly contribute to the development of BM. Of these 5 patients: 4 were ovarian primary cancers, and the samples sent were from metastatic tumors in 3 cases and the primary tumor in 1. The mutations identified in these included cMET in a cerebellar metastasis, TP53 I254S in an omental metastasis and in another brain metastasis sent for analysis the following mutations were obtained: AKT2 amplification, CCNE1 amplification, TP53 E349, BRD4 rearrangement intron 11, and EMSy amplification. 1 of these patients had no target mutation. 1 patient had a primary endometrial cancer, and the BM was sent for mutational analysis. This revealed: EGFR mutation, PD-1, TOP2A and PTEN mutation.

## 6. Rare and Aggressive Histology

Our patient population included patients with small cell, ovarian carcinosarcoma and uterine sarcoma. One of the patients with cervical cancer of small cell histology, was treated for the primary tumor at our institution. This patient was treatment with the SMCC2 protocol that consists of paclitaxel, carboplatin and etoposide followed by concurrent locoregional radiation and cisplatin [18]. She had an excellent response to the primary tumor and was diagnosed with BM 2 years later. She received surgery and WBRT for this. She remains disease free 2 years later todate. One patients with ovarian carcinosarcoma received initial therapy with carboplatin and paclitaxel. In spite of treatment for the primary tumor, this patient was diagnosed with 2 BMs 6 months following initial diagnosis. She received treatment for BM but progressed with intracranial (IC) disease in 6 months. She died 16 months after her initial diagnosis of brain metastasis and had poor quality of life after diagnosis of the IC progression.

## 7. Survival

**Figure 1.**
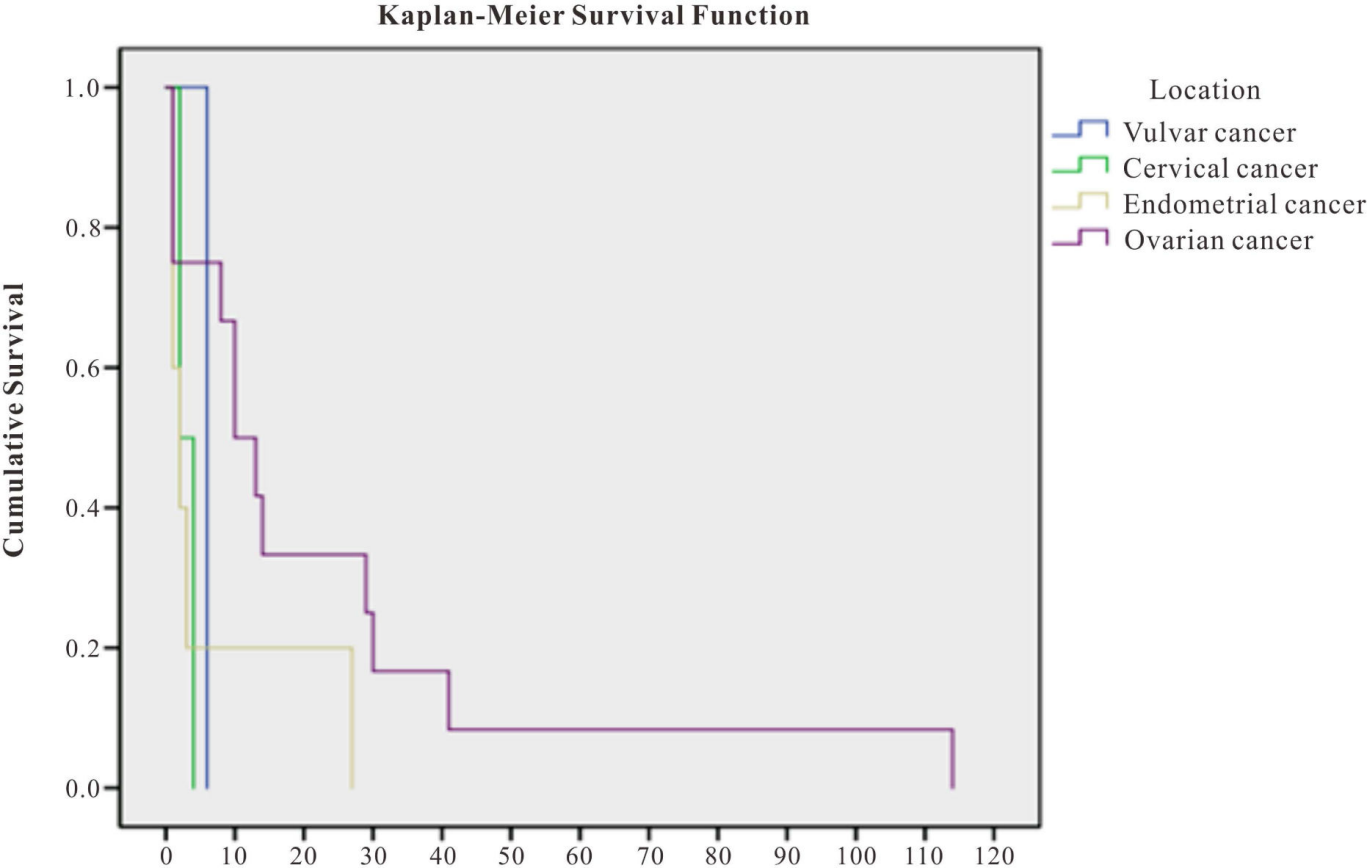
Survival in months from time of the diagnosis of BM to death.

The median overall survival from the diagnosis of the primary tumor was 28 months (6 – 196 months), with a mean of 43 months. Median survival following diagnosis of brain metastasis in patients with ovarian, endometrial, and cervical, was 11.5, 2 and 3 months respectively. There was only one patient with vulvar cancer in our dataset and their survival from diagnosis of brain metastasis to death was 6 months, however, drawing any meaningful conclusions from one case is not advised. Figure 1 illustrates the Kaplan-Meier survival curves that were generated to evaluate survival functions by type of cancer. The primary endpoint in these curves is death. The tests of equality of survival distributions for the different location types was non-significant (log rank test p = 0.153). However, the figure illustrates the trends in probability of survival in months for each type of cancer, with ovarian cancer having the longest probability of survival followed by endometrial. The median time from diagnosis of brain metastasis to death was 9 months (1 month to 9.5 years). The mean survival of patients with Stage I/II primary tumors was 33 months, and the mean survival of patients with stage III/IV primary tumors was 35.3 months. For the Mean survival of patients with 1 BM (10 patients) was 15 months versus 12 months for those with more than 1 BM (13 patients). Those with the largest brain metastasis equal to or >3 cm in size had a mean survival of 13 months versus 17 months for those with <3 cm lesion. The mean survival of patients of RPA class I, II and III was 17.13, 9.64 and 8.87 months respectively.

Those who underwent surgery and radiation, had a mean time from the diagnosis of brain metastasis to death of 32 months. There were 3 patients who received a combination of some form of radiation and chemotherapy without surgery. Their mean survival was 19 months. One patient received surgery, radiation and chemotherapy and this patient’s survival was 12 months.

## 8. Discussion

Although BM has been described as being rare in the literature, it has in fact, with the advent of platinum based chemotherapy, become relatively more common than a decade ago.

Piura and colleagues [4] presented a metanalysis with patients having ovarian cancer and brain metastasis. The studies were all retrospective reviews and dated back as early as early to the mid 1980s. As advances in surgery, chemotherapy and radiation oncology evolved, a trend was seen toward, longer overall survival, but this came with an increased incidence of BM. Mayer *et al*. [6] reviewed autopsy data from five large autopsy series, totaling 576 patients with epithelial ovarian cancer. These series were all published prior to 1978. Collectively they revealed a total of five patients with metastatic disease to the brain as a complication of primary ovarian cancer. Comparing the above data with the data collected in the subsequent years, there clearly is a rise in the incidence of brain metastasis in ovarian cancer.

Our data did not demonstrated that higher grade of the primary tumor had an impact on outcome or survival. This is inconsistent with the literature. Pectasides and colleagues demonstrated in a review that majority of the patients with ovarian cancer that demonstrated brain involvement were of FIGO stage III/IV, and higher FIGO grade [7]. However our numbers are too small to draw definitive conclusions nor can we comment on whether the ability of the cancer to metastasize is related more to the genetic potential of the primary tumor than the grade, or even the stage at which the primary tumor was diagnosed. Lymphovascular invasion and metastatic disease to the lungs are considered poor predictive markers. Again, our numbers were too small to demonstrate this, but patients with these factors did show a shortened survival than those who did not demonstrate these features.

In our study, the presence of extra-cranial disease at the time of diagnosis of brain metastasis, did indicated a shorter survival, which is consistent with the findings of Growden *et al.* whose evaluation of 47 patients with BM in patients with gynecologic cancer proposed presence of extra-cranial disease to be a prognostic indicator [8].

Treatment for BM has evolved over the years and this has led to an improvement in the outcomes of patients in all cancer subtypes, including gynecologic cancers. Cormio and colleagues compared the clinical, pathologic characteristics, treatment and outcomes of patients with ovarian cancer and brain metastasis over 2 sets of time periods: 23 such patients’ charts were reviewed between 1982–1994 and 20 patients between 1995–2000. All patient and pathologic characteristics were similar between the 2 groups but the treatment and their related outcomes were different based on the time period during which they were treated. Patients treated from 1982–1994 received steroids WBRT, WBRT with surgical resection or only steroid treatment. Where as in 1995–2000, 10 patients underwent neurosurgical intervention and received either chemotherapy or radiation therapy alone or in combination, in the adjuvant setting. The 10, who did not get surgery, instead received chemotherapy and/or radiation therapy. The median overall survival of patient treated between 1982–1994 was 5 months. The median overall survival of patients treated between 1995–2000 was 17.6 (p = 0.03) [9].

Our study is similar to a retrospective study from Japan [1]. This was a multi institutional study consisting of 139 patients with all three types of primary tumors. It demonstrated the behavior and prognosis of this patient population and their outcomes following treatment of the metastatic disease to the brain. Patients were recruited over a period of approximately 15 years. They found KPS > 70, single BM, absence of extracranial disease, cranial surgery, cranial radiation and chemotherapy to be independent good prognostic factors. Gressel *et al.* conducted a single institution, retrospective study on 47 patients with gynecologic cancers and brain metastasis diagnosed between 2000–2013. This study demonstrated similar outcomes to what our study suggested, that although BM in the setting of gynecologic cancers had a poor outcome, with multimodality treatment an increase in overall survival was seen [10].

In our study, majority of the patients with BM had ovarian cancer. Patients in our study, who had a low KPS, more than 1 brain lesion, and a high RPA class, had a shorter survival. These findings are consistent with the literature [11] [12], that patient related risk factors are determinants of outcome of radiation treatment and the higher the RPA, the lower the outcome from radiation therapy. There was no difference in survival based on age, stage or CA 125 levels at the time of diagnosis of brain metastasis in our patients. Although our numbers are too small to conclude that multimodality is better than single modality, the trend seen in this study certainly suggest this. Multimodality therapy in this setting, with a combination of surgery, radiation, and chemotherapy, has demonstrated by Anupol *et al*. and Chaura *et al*., in their studies [13] [14].

Tumors of 5 patients were studied for a targetable mutations, no single mutation among the mutations tested was found to be a common mutation in patients with BM. The molecular basis of BM remains to be understood. Ramaswamy and colleagues, through gene expression profiling, found that some primary tumors are pre-determined to metastasize to the brain, and this ability can be detectable at the time of initial diagnosis [15]. This could be helpful in identifying patients with early stage disease who may have a high propensity to metastasize to the brain, and potentially tailor disease specific treatment for such patients sooner, as we have seen from the molecular testing, every patient has their own set of mutations.

We have separately identified our patients with aggressive histology of the primary cancer, such as small cell cervical cancer and sarcoma of the gynecologic tract. There are scarce data on treatment strategies of these tumors, resulting in shorter survival and disease related complications, including BM. These primary tumors are treated differently from the typical adenocarcinoma of the uterus and ovary, and squamous cell carcinoma of the cervix. Small cell carcinoma of the cervix has better outcomes with etoposide-based therapies [16] [17]. For uterinesarcoma, front line therapy at our institution is given with gemcitabine and docetaxel. For recurrent or refractory disease ifosfamide and doxorubicin chemotherapy is used. We have seen durable responses for this nature of disease with these regimens. Trabectadin and pazopanib individually have recently been shown to demonstrate disease control in a disease with a rapid pattern of natural progression [18].

The small number of patients in this study and lack of availability of data on some patients are obstacles to establishing definitive prognostic indicators. There are certain factors that have indicated a trend toward poor survival, including KPS < 70, more than 1 BM, higher RPA class, lack of surgical intervention and possibly histologies such as small cell cancer and sarcoma. The numbers were small to make any comment on the molecular analysis. There was no difference in survival between the various grades or stages of the primary disease.

## 9. Conclusion

There is a need for studies to establish an effective protocol to treat BM from gynecologic primary malignancies. With the advent of new and improved systemic therapies and radiation therapy techniques, BM is becoming a more common entity in the setting of primary gynecologic cancers than a decade ago. Prospective studies to help establish prognostic markers may help us identify early, patients that may be at high risk of BM who may benefit from timely, aggressive therapy. There is a lot to learn from the retrospective studies done to date. In order to conduct informative and beneficial prospective studies, and improve treatment timing and strategy for patients with BM from gynecologic malignancies, incorporation of new tools such as genomic profiling of tumor tissue should be considered.

## Figures and Tables

**Table 1.** Results.

N = 23	Cervical	Endometrial	Ovarian	Vulvar	Total
Total no. of cancers n (%)	4 (17)	6 (25)	13 (54)	1 (4)	24 (100)
Age at original diagnosis mean (sd), range	55 (9.3), 48 – 66	62 (9.1), 49 – 70	60 (10.8), 46 – 79	60 (--)	60 (9.7), 46 – 79
Ethnicity n (%)					
Caucasian	2 (67)	4 (67)	9 (69)	0 (0)	15 (65)
Non-Caucasian	1 (33)	2 (33)	0 (0)	0 (0)	3 (13)
Not described	0 (0)	0 (0)	4 (31)	1 (100)	5 (22)
Histology n (%)					
Adenocarcinoma	0 (0)	3 (50)	9 (69)	0 (0)	12 (52)
Serous type	0 (0)	0 (0)	5 (38)	0 (0)	5 (22)
Sarcoma	0 (0)	3 (50)	1 (8)	0 (0)	4 (17)
Small cell	2 (50)	0 (0)	0 (0)	1 (100)	3 (13)
Squamous	2 (50)	0 (0)	0 (0)	0 (0)	1 (4)
Not described	0 (0)	0 (0)	3 (23)	0 (0)	3 (13)
Stage at diagnosis n (%)					
I	0 (0)	2 (33)	1 (8)	0 (0)	3 (13)
II	3 (75)	0 (0)	0 (0)	0 (0)	3 (13)
III	0 (0)	2 (33)	9 (69)	0 (0)	11 (46)
IV	0 (0)	0 (0)	0 (0)	1 (100)	1 (4)
Not described	1 (25)	2 (33)	3 (23)	0 (0)	6 (25)
RPA class n (%)					
1	2 (50)	1 (20)	7 (54)	0 (0)	10 (43)
2	2 (50)	3 (60)	4 (31)	1 (100)	10 (43)
3	0 (0)	1 (20)	2 (15)	0 (0)	3 (13)
KPS at the time of BM diag. n (%)					
<60%	0 (0)	1 (20)	1 (8)	0 (0)	2 (0)
60% – 70%	3 (75)	2 (40)	1 (8)	0 (0)	6 (0)
71% – 80%	1 (25)	0 (0)	5 (38)	0 (0)	6 (0)
81% – 90%	0 (0)	2 (40)	6 (46)	1 (100)	9 (0)
91% – 100%	0 (0)	0 (0)	0 (0)	0 (0)	0 (0)
Age at BM mean (sd)range	57 (8.7), 51 – 66	63 (9.1), 50 – 72	62 (10.3), 49 – 79	58 (--)	61 (9.3), 49 – 79
KPS at BM mean (sd)range	73 (5.77), 70 – 80	78 (13.30), 60 – 90	82 (9.3), 60 – 90	90 (--)	80.43 (10.22), 60 – 90
Mean time in months (mo)from BM to death (sd)and range	3 (1.4), 2 – 4	7 (11), 1 – 27	23 (31.5), 1 – 114	6	16 (26), 1 – 114
Mean time in mo from primary tumor diagnosis to BM (sd)and range	25 (15), 10 – 39	22 (18), 3 – 45	31 (26), 6 – 82	26	28 (22), 3 – 82
EC progression YES n (%)	2 (67)	2 (33)	3 (23)	N/A	7 (30)
EC progression NO n (%)	1 (33)	4 (67)	10 (77)	1 (100)	16 (70)
